# Main Challenges Expected from the Impact of Climate Change on Microbial Biodiversity of Table Olives: Current Status and Trends

**DOI:** 10.3390/foods12193712

**Published:** 2023-10-09

**Authors:** Antonio Benítez-Cabello, Amélia M. Delgado, Célia Quintas

**Affiliations:** 1 Instituto de la Grasa (CSIC), Food Biotechnology Department, Campus Universitario Pablo de Olavide, Building 46, Ctra, Sevilla-Utrera, km 1, 41013 Seville, Spain; 2 Mediterranean Institute for Agriculture, Environment and Development (MED), Universidade do Algarve, Campus de Gambelas, 8005-139 Faro, Portugal; cquintas@ualg.pt; 3 Instituto Superior de Engenharia, Universidade do Algarve, Campus da Penha, 8005-139 Faro, Portugal

**Keywords:** climate change, table olives, spoilage, safety, mitigation measures

## Abstract

Climate change is a global emergency that is affecting agriculture in Mediterranean countries, notably the production and the characteristics of the final products. This is the case of olive cultivars, a source of olive oil and table olives. Table olives are the most important fermented vegetables in the Mediterranean area, whose world production exceeds 3 million tons/year. Lactic acid bacteria and yeast are the main microorganisms responsible for the fermentation of this product. The microbial diversity and population dynamics during the fermentation process are influenced by several factors, such as the content of sugars and phenols, all of which together influence the quality and safety of the table olives. The composition of fruits is in turn influenced by environmental conditions, such as rainfall, temperature, radiation, and the concentration of minerals in the soil, among others. In this review, we discuss the effect of climate change on the microbial diversity of table olives, with special emphasis on Spanish and Portuguese cultivars. The alterations expected to occur in climate change scenario(s) include changes in the microbial populations, their succession, diversity, and growth kinetics, which may impact the safety and quality of the table olives. Mitigation and adaptation measures are proposed to safeguard the authenticity and sensorial features of this valuable fermented food while ensuring food safety requirements.

## 1. Climate Changes and the Climate Emergency

### Overview of Climate Change on the Iberian Peninsula

Climate change refers to the long-term variation in Earth’s climate patterns and conditions. It is a phenomenon that has raised global concern due to its potentially devastating impact on the environment and life on the planet. Climate change is primarily driven by human activities such as burning fossil fuels, deforestation, and the release of greenhouse gases (GHG) into the atmosphere. The fight against climate change has become a global priority, with efforts focused on reducing GHG emissions, increasing energy efficiency, promoting renewable energy use, and adopting sustainable practices in all sectors of society. These greenhouse gases, such as carbon dioxide (CO_2_) and methane (CH_4_), act as a kind of “blanket” around the Earth, trapping the sun’s heat and increasing the average surface temperature. The imbalance between incoming and outgoing radiation results in global warming. In addition to temperature increase, climate change also leads to disruptions in precipitation patterns, sea-level rise, increased frequency and intensity of extreme weather events, and other harmful effects.

Climate change predictions and assessed impacts are comprehensively enumerated in the IPCC 2023 report [1], from which it can be highlighted that the global annual average temperature is 1.1 °C higher than in pre-industrialization (late 19th century), and large negative impacts are projected for southern regions of Europe [1].

Progressing warming will irreversibly change the composition of terrestrial habitats, increasing in severity as average temperature rises (very high confidence) [1]. WWF, 2018 [2] predicts that if the increase in temperature rises to 2 °C, almost 30% of most species groups are at risk, of which more than a third of all plants. In the “business as usual” case scenario, around half of the region’s current biodiversity will soon be lost. However, while the species we wish to have may go extinct, pathogens and invasive alien species (IAS) are finding conditions to thrive. IAS are one of the biggest causes of species extinction and a global threat to food safety and security, as they spread during extreme weather events such as floods [3].

The Mediterranean region has been pointed out as one of the hot spots of climate change, where substantial warming and drying, and projections of this, show an increase in the likelihood of extreme summer heat and drought, with high confidence levels [1,4]. According to IPCC 2023 [1], climate model predictions point to lower annual average precipitation levels (with medium to high confidence level), although with an increased risk of heavy precipitation and floods. Drought impacts are increasing (observed and projected risks), groundwater reserves are likely to be decreasing, and soil erosion is likely to increase. Drought vulnerability was found to be high, based on data from 1901 to 2010 [1,4].

**Figure 1:**
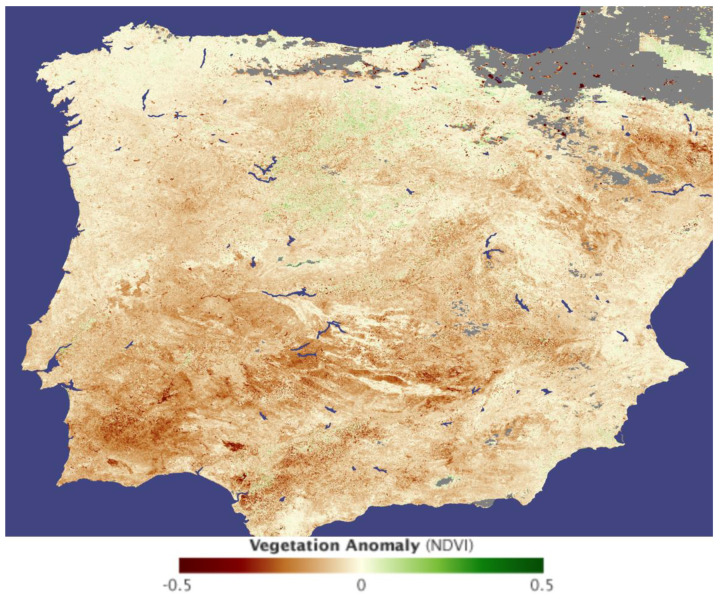
Photo of the Iberian Peninsula showing the impact of the dry weather on vegetation, created using data collected by the Moderate Resolution Imaging Spectroradiometer (MODIS) between 7 April and 22 April 2005. Brown represents those regions where vegetation was thin and less dense than average. Source: NASA Earth Observatory [5].

Water scarcity is an acknowledged problem in the Iberian Peninsula, and the IPCC 2023 [1] report stresses that such risk is likely to increase, causing significant economic losses in water- and energy-dependent sectors. While irrigation can be an effective adaptation option for agriculture, it will be limited by water availability. The drought has been getting worse and worse, especially in the last decade, but was clearly noticeable at least since the early 2000s, as shown in Figure 1.

According to Garrido et al. 2020 [6], when zooming in, this region’s north–south asymmetries are noticeable in time series (1971–2005), with respect to temperature rise and decrease in precipitation levels (annual averages). The coldest temperatures have been registered in the Pyrenees, while the warmest years or seasons (as described by the 95th percentile) were found in the south, and both summer and winter maxima were attained on the southern coast.

EEA 2022 [7] noted that Portugal and Spain were among the countries in the EU with higher values for the average annual drought impact intensity on vegetation in the period 2000–2019. Portugal was in the top position concerning drought impact on cropland productivity, with 61% of croplands affected (7000 km^2^ per year).

IPCC 2023 [1] report argues that key barriers to effective climate adaptation in the area, are limited resources, lack of private-sector and citizens’ engagement, insufficient mobilization of finance, lack of political leadership, and low sense of urgency. The consequences likely include loss of habitat and ecosystem services, crop failures, water rationing during droughts, and loss of soil.

## 2. Influence of Climate Change on Olive Cultivars

### 2.1. Table Olives—Trends in Production and Consumption and Preparation Styles

The olive tree, *Olea europaea L*., has been an icon of the Mediterranean basin since ancient times and has been spreading to other geographic areas. Presently, olive groves occupy approximately 10.8 Mha spread across 58 countries [8], but 97% are still concentrated in the Mediterranean basin. The importance of table olives and olive oil is reflected in the employment it generates. Olive groves employ more than 35 million people worldwide, representing about 1.2% of the world’s active population, and turnover reaches 14 billion euros [9].

The annual world production of table olives reaches approximately 3 × 10^6^ tons/year [10] with Spain, Egypt, and Turkey as the leading producers. In Andalusia (Spain), the olive grove area reaches 1,643,965 ha, of which 6% (98,710 ha) are of varieties destined preferably for table olives. The average production in the last five campaigns was around 5.5 million tons for olive oil production and 474,668 tons of table olives [11].

Portugal is also one of the ten countries with the largest area of olive groves. It ranks ninth, with 361,483 ha of olive groves, representing 3% of the world’s olive grove area. Olive groves and isolated olive trees are found all over the country, but olive plantations are concentrated mainly in Alentejo (50%), Trás-os-Montes (23%), and Beira Interior (14%). The most common table olive varieties in Portugal are Carrasquenha, Cobrançosa, Cordovil de Castelo Branco, Cordovil de Serpa, Galega Vulgar, Maçanilha Algarvia, Redondil, and Negrinha do Freixo [12].

Olive fruits must be processed to eliminate the natural bitterness notably resulting from the presence of oleuropein [13] For this, various processing methods and elaborations are employed. Among the most common techniques for table olives are alkali-treated olives (Spanish style), ripe olives treated with alkaline oxidation (Californian style), and directly brined olives (natural black or green olives) [14]. The green Spanish-style olives, represent approximately 50% of the market, and are highly valued. The corresponding process involves an initial treatment with NaOH (lye), followed by a washing step to remove any excess alkali. Subsequently, the olives are brined, and lactic acid fermentation takes place [13]. In Portugal, most of the table olives are obtained from directly brined olives [15], as mentioned above.

### 2.2. Impact of Climate Change on Biological Conditions of Olive Cultivars—Consequences on Olive Trees and Crop Yields

Globally, olive tree cultivation is approximately limited by 30° to 45° parallels. This latitudinal belt suggests that climatic conditions are a key factor for *O. europaea* development cycle. Olive trees are considered one of the most suitable and best-adapted species for the Mediterranean-type climate, which consists of long, warm, dry summers, alternating with mild cold and rainy winters, mainly corresponding to a Csa type in the Köppen–Geiger climate classification system [16]. This climate classification system, invented in the 19th century by a German climatologist, is under the spotlight because of its holistic nature; it considers biotic factors, such as vegetation [16,17,18].

The challenges faced by olive groves due to climate change are considerable, as the climatic conditions in the region are deviating from their traditional patterns. According to Fraga et al. [19], climate change is significantly impacting olive cultivation. The noticeable effects of climate change, such as rising temperatures and decreasing rainfall, have a detrimental effect on olive groves, primarily through modifications in crop phenology. Crop phenology refers to the relationship between climatic factors and the various stages of crop growth, and it plays a crucial role in olive production. Flowering and maturation are key phases in the olive lifecycle that are particularly susceptible to the influence of extreme weather conditions. Any disruptions to these critical phases can have a decisive impact on olive production.

The increase in temperatures can lead to accelerated phenological development, causing olive trees to bloom earlier and altering the synchronization of flowering with pollinators’ life cycle. This can result in reduced pollination success and subsequent lower fruit set. Furthermore, extreme weather events, such as heatwaves or droughts, cause stress to olive trees, leading to reduced yields and lower-quality fruits. Additionally, the decrease in rainfall poses a significant challenge for olive cultivation, negatively affecting the growth and development of olive trees. Drought stress can also increase the vulnerability of olive groves to pests and diseases. Addressing these challenges requires implementing adaptation strategies, such as selecting resilient olive varieties, optimizing irrigation practices, and adopting sustainable agricultural techniques. Furthermore, coordinated global efforts to mitigate climate change are crucial to safeguard the long-term viability of olive cultivation in the face of changing climatic conditions. Thus, extreme weather events involving unusual temperatures, precipitation, and atmospheric CO_2_ could affect the mentioned variables in the ways described below.

(i) Temperature: The main driver of olive tree phenology by regulating the release from the endo-dormancy period and the flowering state. The transition between the growing and rest period seems to be triggered by temperatures below 14.4 °C (but always positive), while flowering requires the accumulation of “units of cold”. The accumulated exposure to cold temperatures (ideally about 7–9 °C) enables trees to properly set up inflorescence production for springtime when minimum and maximum temperatures start to increase. Thus, when cold requirements are not reached during winter, flowering failures are detected due to the chilling requirements for olive trees, severely impacting olive production [20,21,22]. In the Iberian Peninsula, global warming and the observed trend of increasing minimum temperatures interfere with flowering, and hence with fruit production. Moreover, some olive varieties produce deformed floral buds and fruits under such conditions [23]. According to Dag et al. [24], warmer temperatures and higher evapotranspiration accelerate fruit ripening, which translates into the need for early harvests, though at insufficient maturity of fruits. Moreover, warmer temperatures during olive fruit development will lead to fruits of smaller size, lower pulp/stone ratio and lower maturity index.

**Figure 2:**
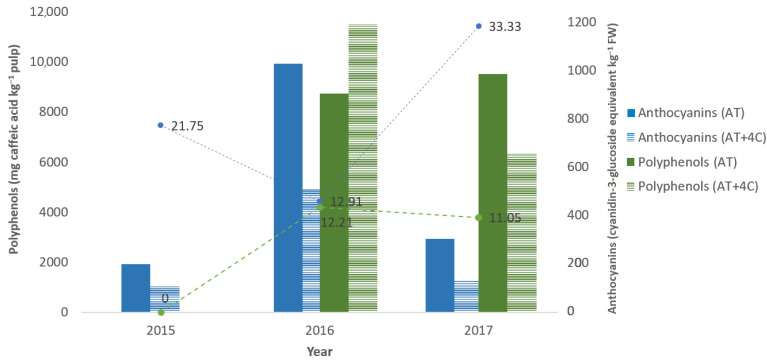
Effect of a 4 °C increase in ambient temperature on fruit anthocyanins (cyaniding-3-glucoside equivalent kg^−1^ FW) and polyphenols (mg caffeic acid kg^−1^ pulp) content. AT: ambient temperature (in 2015, the average ambient temperatures ranged from 7 °C to 30 °C (with a minimum temperature of 1 °C and a maximum temperature of 38 °C), in 2016, they varied between 11 °C and 32 °C (with a minimum of 7 °C and a maximum of 40 °C), and in 2017, they spanned from 8 °C to 30 °C (with a minimum of 2 °C and a maximum of 38 °C)); AT + 4 °C: 4 °C increase in ambient temperature; the average within each column followed by different letters is significantly different at *p* ≤ 0.05 by F-test. The coefficient of variation (%) is represented as dashed lines in green and blue (right axis), where a higher value indicates greater relative variability compared to the mean, whereas a lower coefficient of variation indicates less variability in relation to the mean (Source: [25]).

In a study conducted over three consecutive years, Benlloch-González et al. [25] assessed the effect of temperature increase resulting from climate change throughout the reproductive cycle of the Picual variety. They reported that a 4 °C increase in the environmental temperature reduced fruit yield and affected fruit characteristics and the maturation process. So, in 2015, the average ambient temperatures ranged from 7 °C in winter to 30 °C in summer (with a minimum temperature of 1 °C and a maximum temperature of 38 °C), in 2016, the average temperature varied between 11 °C and 32 °C (with a minimum of 7 °C and a maximum of 40 °C), and in 2017, from the average temperature varied between 8 °C and 30 °C (with a minimum of 2 °C and a maximum of 38 °C). Thus, the accumulation of anthocyanins in fruits during the maturation period showed significant differences between trees exposed to ambient temperatures (AT) and those subjected to a +4 °C treatment (AT + 4 °C) (Figure 2). Higher anthocyanin contents were observed in the fruits of AT trees in all seasons; in other words, warmer temperatures during this period reduced the accumulation of anthocyanins in the fruits of AT + 4 °C trees. There were no differences in fruit polyphenol contents between treatments in 2016. However, the high-temperature treatment decreased fruit polyphenol content in 2017 (Figure 2). The coefficient of variance exhibited greater variability in the anthocyanin dataset, with the highest value recorded in 2017 at 33.33%. In contrast, the polyphenol dataset displayed its highest coefficient of variance in 2016, reaching a value of 12.21%. This indicates that the data for anthocyanins demonstrated more significant fluctuations over the study period compared to polyphenols.

(ii) Precipitation: *O. europaea* is a xerophyte plant, tolerant to water stress but well adapted to rainy winters because Mediterranean soils are able to retain water, even if poorly. Olive growth and economic yield are compromised when annual precipitation is lower than 350 mm/year [19]. Even in irrigated orchards, growers will depend on the efficient use of winter and spring rainfall for productivity. On the other hand, with the occurrence of short but heavy rainfalls during warm periods, the incidence of pests and diseases is also expected to increase in addition to soil erosion, not to mention potentially negative synergies impacting yields.

(iii) Atmospheric CO_2_ concentrations: increased CO_2_ concentrations have positive effects on crop productivity. CO_2_ is an input for photosynthesis, and studies have shown that high CO_2_ levels can enhance the efficiency of water use by crops through a reduction in stomatal conductance [26]. This phenomenon is attributed to the stimulation of photosynthesis and the resulting decrease in transpiration rates. The improved water use efficiency by CO_2_ has significant implications for agricultural productivity, particularly in water-limited regions. Crops can maintain their growth and yield while minimizing water loss through transpiration. This increased efficiency allows for more sustainable use of water resources in agriculture. However, it is important to note that there is still considerable uncertainty regarding the compensatory effects of increased CO_2_ levels in the context of future climatic conditions [27]. Climate change introduces complex interactions between CO_2_, temperature, precipitation patterns, and other environmental factors that can influence crop growth and productivity. While elevated CO_2_ levels may enhance water use efficiency, they may not fully offset the potential negative impacts on other variables. Future studies will play a crucial role in providing insights into how crops respond to combined changes in CO_2_ levels and climatic variables. By gaining a deeper understanding of these interactions, we can develop more accurate predictions and strategies for sustainable agriculture in the face of a changing climate.

**Figure 3:**
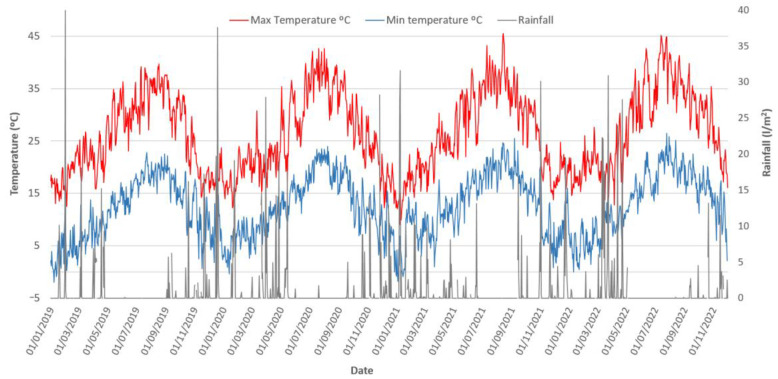
Daily values of maximum (red) and minimum (blue) temperature (°C) and rainfall (grey) (L/m^2^), measured in the Arahal region (Seville, Spain) over four years (2019–2022) [28].

Figure 3 shows data on maximum and minimum temperatures and daily rainfall over the last 4 years at Arahal [28], one of the main table olive production areas in Andalusia, Spain. In this area, the average temperature has been increasing progressively (Figure 3). In this way, over a period of 4 years, an increase in the maximum temperature of 5.5 °C has been observed, reaching maximum values in 2022 of 44.7 °C and a minimum of 22.9 °C in July 2022 compared, respectively, to 39.2 °C and 19.1 °C in 2019 in the same month. This suggests that nights may be becoming warmer, which could have consequences for the physiology of the plants and the development of the olives. Similarly, a progressive decrease in rainfall was observed throughout the same study period. Thus, while in 2019 the maximum rainfall was 40.4 L/m^2^ in January, in 2022, only a maximum value of 13.2 L/m^2^ per month was reached. This decrease in rainfall could have a negative impact on water availability for irrigation and the healthy development of olive trees. Taken together, these results suggest a change in climatic conditions that could be detrimental to table olive production. High temperatures and the lack of precipitation may lead to drought conditions and water stress, which in turn affect olive productivity and, ultimately, table olive production. Adaptation to climate change is becoming increasingly important in agriculture to ensure the security and sustainability of food production, beyond the olive sector.

**Figure 4:**
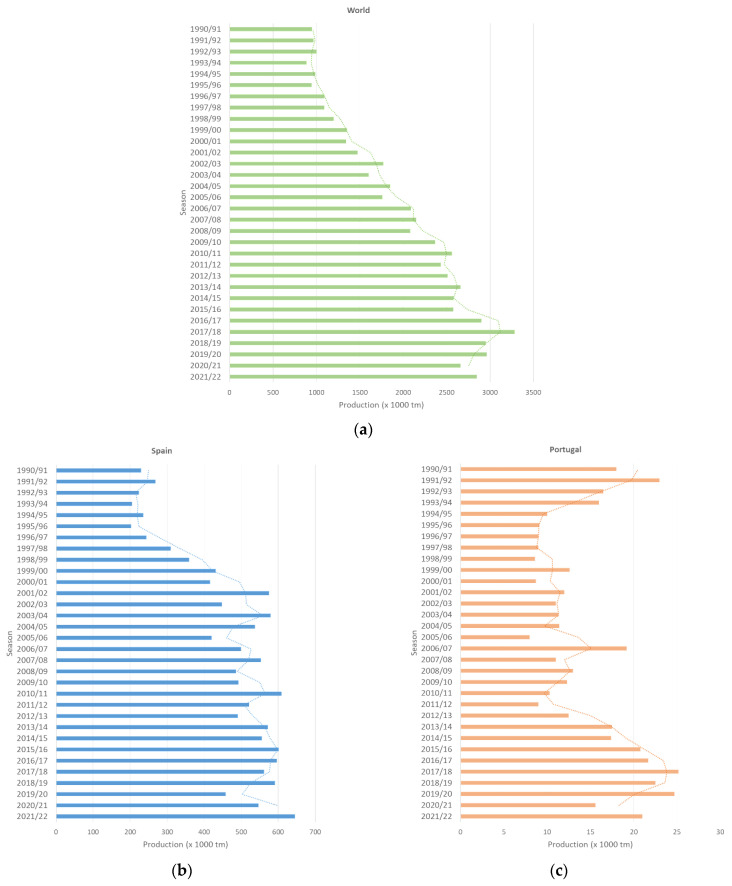
Table olive production (tons) from 1990 to 2022. (**a**) World production; (**b**) Spanish production; and (**c**) Portuguese production. The dashed line refers to the production trend.

If we take into account the world’s average production of table olives over the last 32 years (Figure 4), it can be seen that production has been increasing drastically as a result of the demand and improvement in production methods. When analyzing the provided data, it can be observed that olive production has been declining since 2014/2015. Before the 2014/2015 campaign, olive production exhibited some variability, with increases and decreases in different periods. However, from 2014/2015 onwards, a more pronounced decrease in global production can be noticed. A similar pattern is observed in recent years in Portugal. When inspecting the data more closely, we found that in Spain, this slowdown occurs much earlier. Production seems to stabilize from the year 2000 onwards, which may be due to the increase in temperature and decrease in rainfall in the main production areas.

On the other hand, it is also important to consider that the decrease in production may be offset by an increase in irrigation systems, which has been growing over the years. According to a survey on Crop Surfaces and Yields in Spain (Esyrce) 2021, published by the Ministry of Agriculture, Fisheries, and Food, irrigated olive groves in Spain grew by 22.58% in 2021 [29]. Nevertheless, despite this, and other implemented measures, such as the conversion to dryland olive groves, there has been a noticeable trend of declining production in recent years.

(iv) Other effects: the increased salinity of the soils is a condition observed in various regions where precipitation is gradually decreasing, namely in the Southern Iberian Peninsula. In addition to the effects on the tree’s physiology, low precipitation levels alongside the same usage pattern of chemical fertilizers may lead to an increasing concentration of certain salts in the fruits, later impacting the fermentation. For example, the increase in potassium in the fermentations may change the main microbial species involved, according to the study of Mateus et al. (2016) [30]. The changes in the mineral composition of soils and waters/brines may affect the diversity of fermentative microorganisms, with consequences, if not at the safety level, certainly for the final product’s organoleptic quality [31,32].

### 2.3. Impact of Climate Changes on the Main Chemical Compounds of Olives

Valente et al. (2020) [33] studied the effect of drought and heat conditions on the fruits of the Portuguese varieties Cobrançosa, Cordovil de Castelo Branco, and Cordovil de Serpa. According to the results, those stresses modify the metabolite profile of olives in different ways, depending on the cultivar. After a drought and heat shock, Cobrançosa showed the highest levels of oleic, cis-vaccenic, and palmitic acids. Stressed olives of this cultivar increased their level of oleic acid by 58%, compared to the control. In the other cultivars studied, the stress caused a reduction in the levels of cis-vaccenic and stearic acids, and had a tendency to lower the ratio of mono-unsaturated fatty acids (MUFA) to saturated fatty acids (SFA) (MUFAs/SFAs) compared to the control, contrary to the Cobrançosa olives. In the case of Italian varieties, water shortage diminished the content of unsaturated fatty acids in the cultivars Cipressino [34], Frantoio, and Leccino [35], also pointing to the idea that different cultivars respond differently to environmental pressures.

Regarding organic acids, Cobrançosa exhibited the highest levels of malic, citric, and quinic acids, and these levels remained unaffected by stress. However, these conditions led to an increase in the content of the same acids in the case of Cordovil de Serpa and led to a decrease in Cordiovil de Castelo Branco. The richness of Cobrançosa in these acids may indicate that this cultivar can naturally cope better with oxidative stress arising during heat/drought events, as reported by Dias Correia et al. (2018) [36]. Among the cultivars, Cobrançosa, Cordovil de Castelo Branco, and Cordovil de Serpa, Cobrançosa is the most tolerant to drought and heat episodes [36].

Regarding soluble sugars and polyols, the exposition to drought and heat resulted in an accumulation of these compounds (glucopyranose, furanoses, and sorbitol) in the olives of all the studied cultivars.

In regard to phenolic compounds, Cobrançosa stressed fruits presented contents of hydroxytyrosol and oleuropein lower than the control, but showed an increased level of p-coumaric acid. In the case of Cordovil de Serpa, the secoiridoids, flavonoids, and cinnamic acid decreased after the environmental stress. Conversely, stressed Cordovil de Castelo Branco olives exhibited a higher oleuropein content but lower flavonoid levels. Water deficit has been reported to increase the accumulation of phenolic compounds in olives [37,38], but this was not observed in Cobrançosa or Cordovil de Serpa olive varieties.

## 3. Impact of Biological Changes on Table Olive Microbiological Populations

### 3.1. Microorganisms Involved in Table Olive Fermentations

The most commonly identified microorganisms responsible for the fermentation process are mainly lactic acid bacteria (LAB) of the *Lactiplantibacillus* (ex *Lactobacillus*) genus, which convert sugars to lactic acid and lower the brine’s pH. They coexist with yeasts that provide interesting technological features, with *Pichia*, *Saccharomyces*, *Candida*, and *Wickerhanomyces* as the most relevant genera [39,40,41,42]. Both, LAB and yeast determine the flavor, quality, and safety of the final product [13,41]. It has been generally established that LAB are responsible for the fermentation of treated olives. LAB and yeasts have been noted to coexist in the fermentation of untreated olives (naturally brined). In some cases, yeasts can be exclusively responsible for the fermentation of untreated olives [30,43].

However, during fermentation, spoilage and harmful microorganisms can also grow (*Enterobacteriaceae*, *Clostridium*, and *Propionibacteriaceae*), leading to product deterioration and the appearance of unwanted organoleptic characteristics [13,44,45,46]. In fermentations depending on epiphytic and environmental microbiota, a mixed population of LAB and yeast is expected to overgrow the spoilage and pathogenic bacteria, which are less adapted to brine’s composition, notably to the high concentrations of NaCl and phenolic compounds. LAB and yeast change brine’s composition by producing weak acids and other compounds. Besides lowering the pH, weak acids (e.g., lactic acid) are bacteriostatic *per se* [47,48]. Many of those LAB and yeast have been shown to inhibit direct competitors by releasing inhibitory compounds against them, as is the case of LAB bacteriocins. The relevance of these antimicrobial peptides in the control of table olive fermentations has been noted for some time [49,50,51].

On the other hand, the emergence of New Generation Sequencing techniques and Omic sciences has unveiled previously unknown biodiversity in table olive fermentations that could not be described using traditional culture-dependent techniques. This is exemplified by metataxonomic studies, which became an essential tool for studying microbial biodiversity during fermentation. These studies revealed the presence of various bacterial genera such as *Alkalibacterium*, *Marinilactibacillus*, and *Salinibacter*, among others, whose specific role in the fermentation process remains unclear [52,53,54,55].

### 3.2. Impact of Climate Change on Fruit Composition, Fermentation, Microbial Population, and Product Safety

The ultimate goals of the fermentation process are, on the one hand, to preserve the nutrients of the olive, and, on the other hand, to improve the organoleptic properties of the final product [56]. Several factors will influence the correct development of fermentation and must be evaluated to control the ecosystem [57,58,59,60,61]. Thus, the olive ecosystem is influenced by the indigenous microbial population, by the intrinsic factors related to the olives (pH, a_W_, phenols, sugar content, etc.), and by extrinsic factors (temperature, oxygen, or (NaCl concentration). Both extrinsic and intrinsic factors will determine the diversity of microorganisms during fermentation; many of them, in turn, will be influenced by climate change conditions.

Among the main factors that influence microbial growth during fermentation is the sugar content of the fruit. Olives contain various fermentable carbohydrates that generally range from 2.6% to 6% of the olive content [62]. However, when the olives are washed or lye-treated, sugars are lost along with other soluble compounds. Olive fermentation is considered to be over when the sugars are totally consumed by microorganisms.

The consumption rate of the sugars remaining in the olive flesh can vary depending on the microorganisms and specific fermentation conditions. Generally, the sugar consumption follows a specific sequence. Thus, glucose and fructose are the most easily fermentable sugars and are rapidly consumed by the LAB and yeast [63]. They are converted into lactic acid and ethanol, respectively. After the reducing sugars are consumed, sucrose is broken down by microbial sucrases, and the resulting glucose and fructose are then metabolized by LAB and yeast. Maltose, another disaccharide composed of two glucose molecules, can be consumed by some strains able to produce maltases for its breakdown [63]. Other less abundant sugars, such as mannitol and xylose, may be consumed to a lesser extent by certain microorganisms during the fermentation [64,65,66]. It is important to note that the order in which sugars are consumed varies with temperature and pH during the fermentation, as well as with the presence of different microbial species/strains. Also, the sugar content in the raw product can be affected by several variables in the field, such as ambient temperature and rainfall, as well as the cultivar and maturation stage, among others.

**Figure 5:**
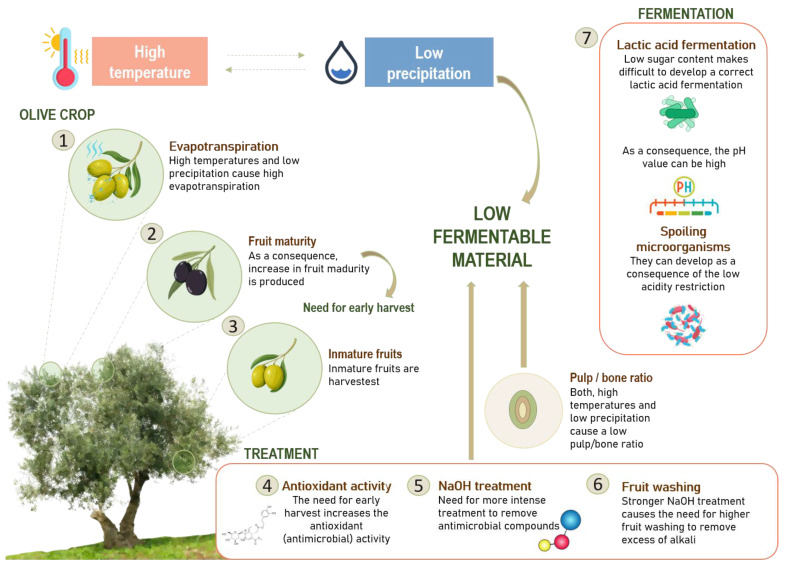
Projected impact of high temperatures and low rainfall on microbial populations in Spanish-style table olives.

Figure 5 illustrates the projected impact of climate change, specifically high temperatures and low rainfall, on the growth of microbial populations in Spanish-style olives. The data demonstrate a steady rise in average temperatures over the years, especially in the months leading up to the harvest season. These warmer temperatures, taken together with the reduced rainfall, can result in increased evapotranspiration in olive trees (1). As a result of increased evapotranspiration, excessive maturity, characterized by a high-fat content but a low sugar content, could be promoted (2), which hampers the development of subsequent lactic fermentation. This necessitates an early harvesting to prevent fruit overripening. However, early harvesting may lead to the collection of immature fruits (3), resulting in elevated antioxidant (antimicrobial) activity within the fruit (4). Phenolic compounds, such as phenolic acids, phenolic alcohols, flavonoids, and secoiridoids, are crucial in table olives. Notably, compounds like oleuropein and its hydrolysis derivatives exhibit antimicrobial properties against various microorganisms, including LAB, with the exception of a few strains, able to use the sugar moiety from secoridoids [67]. The inhibitory effects of many polyphenols on LAB growth have been extensively researched. Furthermore, an increase in oleuropein content in the growth medium reduces the ability of bacteria to hydrolyze this glycoside. This, in turn, poses a challenge to the fermentation process. Consequently, more aggressive NaOH treatments involving higher concentrations and durations become imperative to eliminate antimicrobial compounds and facilitate the fermentation process (5). These treatments soften the fruit pulp and result in a high residual NaOH content, leading to an initial fermentative pH above 10. Thus, it is worth noting that higher NaOH treatments require additional washing steps to remove excess alkali (6). Consequently, the combination of strong NaOH treatments and extensive fruit washing, at the processing level, with reduced precipitation, at the field production level, collectively diminish the availability of fermentable nutrients. Furthermore, both high temperatures and low precipitation levels contribute to a decreased pulp-to-bone ratio, which, in turn, impacts the sugar available within the olives’ flesh. An adequate sugar content is critical for the successful progression of fermentation. Low sugar contents in the fruit complicate the achievement of proper lactic acid fermentation, leading to inadequate pH values. Low pH is crucial to inhibit the growth of spoilage microorganisms such as certain *Enterobacteriaceae*, *Propionibacterium*, or *Clostridium* (7).

The pH and free acidity data gathered from the fermentation of Spanish-style table olives over the past four campaigns (2019–2022) in two distinct industries in Seville provides compelling evidence that supports the projected scenario depicted in Figure 5. Table 1 demonstrates a consistent rise in pH levels observed throughout the fermentation process across the various campaigns. This increase can be attributed to a decrease in the production of lactic acid, which is reflected in the obtained values of free acidity. Thus, the table clearly displays a strong correlation between the increase in temperature and the progression of lactic acid fermentation.

Throughout the four-year study, a clear upward trend in the average ambient temperatures has become evident. This rise is also reflected in the increasing minimum and maximum temperature values. Although the evolution of average rainfall during this period is not as pronounced, we observed a decreasing tendency in the maximum rainfall from 2019 (40.4 L/m^2^ in January) to 2022 (13.2 L/m^2^) (Figure 3). This shift towards drier and warmer conditions may have a notable impact on fermentation processes towards increased pH and reduced levels of free acidity. Considering the information presented above, these changes can be attributed to shifts in the dominant microbial populations throughout the fermentation process. These consequences include the displacement of previously common epiphytic microbiota and environmental microorganisms, such as LAB and yeast, by less desirable groups like *Enterobacteriaceae* and filamentous fungi. These microbial alterations can lead to inferior sensory characteristics and higher food safety risks.

#### 3.2.1. Microbial Origin Alterations in Table Olives

The difficulty in the development of lactic fermentation as a consequence of climate change can pose challenges and lead to the growth of spoilage microorganisms. One of the most significant issues within the industry is known as “Alambrado”. This particular type of alteration is characterized by a line in the fruit’s pulp, and has been attributed to the activity of certain microorganisms, particularly Gram-negative bacilli such as *Enterobacter*, *Citrobacter*, *Klebsiella*, *Escherichia coli*, and *Aeromonas*. These microorganisms consume the sugars present in the fruit and produce gases, including CO_2_ and H_2_, through their fermentative activity. Over time, these gases accumulate and form gas-filled pockets or sacs, which destroy the pulp and separate it from the fruit’s skin. Alambrado is often associated with prolonged fermentative pH values higher than 4.5 units.

In a recent study by de Castro et al. (2022) [68], *Celerinatantimonas* was identified, for the first time, as a crucial microorganism responsible for gas pocket formation. Subsequently, Ruiz-Barba et al. (2022) [69] investigated the main factors influencing the growth of *Celerinatantimonas* sp. and gas pocket formation in Spanish-style green olives. Their findings indicated that pH played a critical role, with growth occurring within a pH range between 4.5 and 7.5. To prevent that defect and inhibit the growth of *Celerinatantimonas*, it is necessary to maintain the pH below 4.5 at the beginning of fermentation, which can be achieved by adding lactic acid as recommended by Ruiz-Barba et al. (2022) [69] to effectively control *Celerinatantimonas* growth and mitigate the gas pocket defect.

In the case of natural table olives, low pH values are also considered a keystone of the microbial quality of table olives, according to the International Olive Council [70]. However, often a tendency towards higher pH values is observed and polyphenol-resistant yeasts tend to predominate. Consequently, the risk of defects of microbial origin should be considered, especially at the initial steps before the dominance of the desirable fermentative populations. While the growth of aerobic bacteria would be limited by warranting anaerobic conditions, uncontrolled growth of LAB or spoilage yeast, such as *Zygotorulaspora* sp., may directly lead to texture and flavor deviations [71].

In addition, microbial stability in completed fermentations might also be challenged at higher pH values, especially if low acidity accompanies this phenomenon or if residual sugars remain in the fermented brines. It is worth noting that the quality of natural table olives is only guaranteed when the packed brine has a pH value lower than 4.3, an acidity higher than 0.3 g lactic acid/100 mL, and a minimum of 6 g NaCl/100 mL [70]. In the case of naturally fermented olives, those pH and acidity values may not be achieved at the end of fermentation, limiting their shelf life and suitability to be packed. Arroyo-López et al. (2009) [72] and Alves, et al. (2015) [71] reported that in Manzanilla-Aloreña and Mançanilha Algarvia cultivar olives, the microbial activity during the shelf life may contribute to the production of gas and swelling of the containers. In fact, in the case of fermented food production, such as that of table olives, some yeast species can be attributed positive functions and negative roles, as discussed by Arroyo-López et al. (2008) [73]. The line between advantageous fermenting and degradation metabolic routes (especially with yeasts) is hard to define.

In addition, the uncontrolled growth of filamentous fungi, eventually present in the fruits and brines, may cause the degradation of flavor and texture due to the production of extracellular enzymes, as well as secondary metabolites. Among those, mycotoxins should be highlighted because they pose serious health problems (carcinogenic, hepatotoxic, nephrotoxic, and neurotoxic). The production of mycotoxins has been associated with the global increase in temperature and its impact was reported in winemaking [74]. In our view, it may also occur, and even be more frequent, in other fermented products. Special attention should be paid to “emerging mycotoxins” (toxins for which little knowledge is available), such as those produced by members of the genus *Fusarium*, namely enniantins [75], *Alternaria*, namely alternariol [76], and others. The increased probability of the occurrence of emerging and better-known mycotoxins in table olives should be considered in the face of climate change scenarios, as fungi easily adapt their biosynthetic pathways [77] and have been developing thermotolerance [78].

Biogenic amines, formed by the decarboxylation of certain aminoacids, such as lysine, which turns into cadaverine, are another class of microbial compounds of concern. They are most often associated with the uncontrolled growth of some LAB, such as *Leuconostoc* sp., *Enterococcus*, and *Enterobacteriaceae* [79,80]. Biogenic amines pose safety concerns, and their occurrence in table olives can be more frequent as a consequence of microbial adaptation mechanisms to climate change [81].

Because olive fermentation is still craft-based, it is not fully predictable, and some alterations can occur. During the first phase of Spanish fermentation, the Gram-negative bacteria prevail. This phase lasts until LAB increase in number and induce a decrease in pH. If this reduction is not too fast, “gas pockets”, resulting in the softening and breakage of the cuticle, can appear. A high pH can also favor the development of *Clostridium* spp., which could induce putrid or butyric fermentation, resulting in off-flavors and off-odors. The softening of the olive drupe is another alteration due to the development of pectinolytic yeasts (e.g., *P. manshurica*, *Pichia kudriavzevii*, *Saccharomyces oleaginosus*, etc.), molds (*Aspergillus niger*, *Fusarium* spp., and *Penicillium* spp.) and some bacteria (*Bacillus* spp., *Aerobacter* spp., etc.). These microorganisms release degrading enzymes, which act on pectic substances and cellulose, hemicellulose, and polysaccharides, causing the loss of the structural integrity of the olive drupe. Seville-style table olives can undergo a defect called “white spot”. These spots develop between the skin and the flesh and are associated with the development of some *L. plantarum* strains. Finally, when the final product is not pasteurized, *Propionibacterium* can develop, producing acetic and propionic acids. This alteration is called “zapateria” and causes an increase in volatile acidity and the formation of cyclohexane carboxylic acid and the production of biogenic amines, such as cadaverine and tyramine [14].

#### 3.2.2. Effect of Temperature during the Fermentation

Controlled temperatures during the fermentation steps are generally beneficial, especially in those regions where the fermentation temperature follows environmental fluctuations. Unfortunately, in most companies, temperature control is not applicable because it requires an expensive procedure. Tassou et al. (2002) [61] revealed the importance of temperature in the fermentation processes. Although they ended up inhibiting Gram-negative bacteria, an initial increase in the population of Gram-negative bacteria, such as *Pseudomonas* and *Enterobacteriaceae*, in brines of naturally fermented Conservolea table olives at 25 °C and 18 °C at ambient temperatures (7–15 °C), was observed. In addition, they reported that the highest death rate occurred in fermentations at room temperature, also showing less growth of LAB. Therefore, an increase in temperature can favor both, whose predominance will depend on other variables, such as salt concentration or pH. In this case, they observed that yeasts were the predominant group in fermentations at room temperature, indicating a lower sensitivity to temperature increases.

Rodrigues et al. (2010) [82] studied the effect of different processing treatments (Process A—olives submitted to washing treatments before brining; Process B—olives immediately brined) on the variation of physicochemical and microbial parameters during the fermentation of cracked green table olives at two temperatures (25 °C and 18 °C). The final values obtained are summarized in Table 2.

The initial brining conditions affected the pH values, reducing sugars, phenolic contents, and levels of microorganisms in the final brines (Table 2). The *Enterobacteriaceae* group was present in the final brines of pre-washed olives, fermented at 25 °C but not in the olives immediately brined. At 18 °C the *Enterobacteriaceae* group was not detected in the final fermentation brines. The loss of acids and phenolics resulting from the washings give origin to fermentation processes where enterobacteria found conditions to grow at 25 °C, which does not happen at 18 °C. In case of a decrease in phenolic compounds, natural table olives processed at 25 °C are subjected to increased risks of spoilage and safety of the final product. At higher temperatures, the time required for fermentation is lower and, if not controlled, may lead to the production of off-flavors, as well as the growth of several potential types of food spoilage that may also pose food safety risks.

On the other hand, the type of fermenter used will also directly influence the temperature. The smaller the fermenter, the less inertia it has. For example, in fermentations occurring in 200-L barrels, a very irregular growth of the microbiota could develop, posing challenges for adjustments. In contrast, the use of multiple smaller barrels can yield a mixed outcome, where some may suffer damage or spoilage, while others may contain exceptionally high-quality olives [83].

**Table 2:** Microbial and physicochemical characteristics of fermentative brine after 30 days of fermentation and microbiological counts of unprocessed fruits (A—olives submitted to washing treatments before brining; B—olives immediately brined) (ND—not determined) [84].

Microbial and Physicochemical Characteristics		Total Microbiota Log CFU/mL	Yeasts Log CFU/mL	Enterobacteria Log CFU/mL	pH	Reducing Sugars g/L	Total Phenolic Compounds g Gallic Acid/L
**Fermentation** **Process**	**Unprocessed fruits**	4.08 ± 0.30	3.33 ± 0.18	<Detection limit	ND	ND	ND
**Process A**	25 °C	7.16 ± 0.65	6.47 ± 0.17	6.49 ± 0.21	4.31 ± 0.03	0.76 ± 0.07	0.28 ± 0.01
**Process B**		6.46 ± 0.06	6.15 ± 0.11	<Detection limit	4.54 ± 0.00	2.89 ± 0.41	0.89 ± 0.00
**Process A**	18 °C	4.93 ± 0.02	4.97 ± 0.19	<Detection limit	4.31 ± 0.10	1.97 ± 0.33	0.29 ± 0.02
**Process B**		5.15 ± 0.11	5.16 ± 0.31	<Detection limit	4.51 ± 0.04	12.31 ± 1.37	1.09 ± 0.07

Plants adapt to environmental changes by adjusting secondary metabolite pathways. On the other hand, microbial populations can adapt faster because of their very short lifecycles (or generation time). Therefore, we have to be able to predict and mitigate issues related to reduced fruit yields, product quality, and food safety, as detailed in Table 3 and Table 4.

Spoilage microorganisms pose significant threats due to potential transfer of pathogenicity, microbial resistance, and other genetic elements. Table 3 and Table 4 provide a list of undesirable microorganisms previously detected in table olives, which are susceptible to growth under uncontrolled conditions, including high temperatures resulting from climate change.

It is important to highlight that the physicochemical and microbiological features achieved by the fermentation process of table olives make it a safe product because it results in conditions unsuitable for the development of spoilage and pathogenic microorganisms (e.g., low pH, no sugars, etc.). However, numerous research studies indicate that it is not exempt from risk.

Climate change can have significant effects on the development and proliferation of such microorganisms in table olives. The rise in ambient temperature can facilitate the growth of thermophilic bacteria like *Campylobacter*, which thrives between 20 °C and 45 °C, or of mesophilic bacteria, like *Bacillus cereus* or *Staphylococcus* spp., which have been described as part of the olive’s microbiota during the initial fermentation process. Under normal fermentation conditions, it is unlikely that certain bacteria will be present or multiply significantly. The brine used during fermentation, with its high salt content, acts as an inhibitor of the growth of pathogenic bacteria. However, it is important to note that the quality and safety of table olives can depend on hygiene and handling practices throughout all stages of production, from olive harvesting to final packaging. Unfortunately, these practices are not always guaranteed, and numerous studies have identified undesirable microorganisms in the final products, whose growth may be favored by rising temperatures. For example, *Listeria monocytogenes* has been found in marketed table olives in Europe, including Spanish-style processed green Nocellara dell’Etna olives [99,100]. Furthermore, microorganisms can adapt to the conditions imposed by climate change. This adaptation can impact the effectiveness of traditional control practices, necessitating constant surveillance and adaptive measures to ensure food safety. *Listeria monocytogenes* is a prime example, as its survival and ability to form biofilms are favored by high temperatures. Additionally, this bacterium has the ability to grow at refrigerated temperatures, making it an ongoing problem for the food industry and consumers during long-term storage, even at refrigeration temperatures.

The increase in temperature during table olive fermentation can also promote the growth of *Clostridium* sp. Table olive fermentation typically occurs at an optimal temperature range of 20 to 30 °C. However, the previously shown data (Table 1) indicate an increase in the maximum temperature of 5.5 °C over the last 4 years, reaching a maximum value of 44.7 °C in 2022. When we focus on the temperature data for the months of September to November, which coincide with the fermentation process in the specified regions, we see that in 2022, the temperature range ranged from 39.8 °C to 35.4 °C. This range falls within the optimal growth temperature range for proteolytic strains of *Clostridium*, which are known to thrive best between 37 °C and 42 °C. These strains also have a minimum pH value, limiting their growth at pH 4.6. Therefore, prolonged exposure to temperatures above this range, along with the described pH values, can promote the growth of this microorganism. The same applies to *Staphylococcus*, whose growth ranges from 7 °C to 47.8 °C, with 35 °C as their optimum, and a pH range between 4.5 and 9.3 (optimum 7.0–7.5). Consequently, there is a higher likelihood of these microorganisms developing during the initial stages of fermentation if the pH values are not corrected.

On the other hand, the presence of bacteriophages in fermented vegetables is common. In this regard, although there are few studies dedicated to the identification of bacteriophages in table olive fermentation, there is evidence of their presence [110], and current studies point in this direction. Bacteriophages are viruses that infect bacteria, regulating their population. Lanza et al. [110] detected the presence of phages in olive fermentations that infected strains of *L. plantarum*, the main microorganisms, along with *L. pentosus*, responsible for the fermentation process. This evidence may have implications for the safety of the final product and the development of the fermentation process. Although bacteriophages are viruses that infect bacteria and are not harmful to humans, their presence can affect the fermentation process and, in some cases, compromise the quality and safety of the final product. It has been shown that they play an important role in bacterial evolution and diversity. By infecting the bacteria involved in olive fermentation, they can reduce fermentative activity. This can result in a lower production of lactic acid, which is responsible for the preservation and proper acidity of the final product. Additionally, the presence of bacteriophages can lead to the development of unwanted bacteria during fermentation, which can affect the taste, texture, and quality of the final product [111].

Studying how climatic conditions affect phage development is an aspect to be investigated. However, studies indicate that high temperatures and bacteriophages can indirectly select bacterial pathogenicity in environmental reservoirs [111]. The researchers conducted experiments using the pathogenic bacterium *Pseudomonas fluorescens* and its specific phage. They exposed the bacteria and the phage to different temperatures and then evaluated changes in bacterial pathogenicity. The results of the study showed that high temperatures and the presence of bacteriophages can influence bacterial pathogenicity. In particular, it was observed that high temperatures increased the virulence of the bacteria. Additionally, the presence of bacteriophages also had an indirect effect on the selection of bacterial pathogenicity, as bacteriophages promoted the spread of more virulent strains of bacteria. These findings suggest that environmental changes, such as temperature variations, and the interaction between bacteriophages and bacteria can play an important role in the selection of pathogenic bacteria in environmental reservoirs [111].

On the other hand, *Pseudomonas* species are known for their versatility in adapting to various environments, which is primarily attributed to their diverse metabolism. They can utilize a wide range of carbon sources, including contaminants like hydrocarbons, and possess alternative pathways for energy generation. This metabolic adaptability is crucial in the context of table olive fermentation [86,87,108,120,121]. Temperature plays a significant role in the growth of *Pseudomonas* species. In laboratory conditions, most of these bacteria thrive between 25 and 30 °C. However, some strains exhibit adaptability to both warm and cold environments, with growth occurring between 0 and 45 °C, depending on the species. For instance, *P. aeruginosa*, grows optimally at 37 °C, can survive at 42 °C, and can grow at 15 °C, but not below 10 °C [123]. The temperature sensitivity of *Pseudomonas* species has implications for their interaction with host organisms, and could be influenced by climate change. Furthermore, the increase in temperature due to global climate change could trigger outbreaks of pathogens like *Pseudomonas*. Rising temperatures, linked to global climate change, may impact bacterial–host interactions, potentially leading to shifts in disease patterns [122]. Mathematical models have been developed to predict the distribution and virulence of pathogens, such as *P. syringae pv. actinidiae* in kiwi plants, showing temperature-dependent redistribution and changes in virulence [126]. Also, studies have investigated the formation of biofilms in water pipelines. An increase in temperature from 16 °C to 24 °C has been found to promote the growth of *P. aeruginosa*. This could lead to health concerns in systems where *Pseudomonas* biofilms develop [127].

Understanding the consequences of global warming on microbial physiology in natural cycles like table olive fermentation remains a challenge for researchers. The ability of some bacteria to adapt to changing temperatures and their potential impact on various ecosystems underscores the importance of further study in this area. However, it is necessary to indicate that the development of spoiling or pathogenic microorganisms, such as *Pseudomonas*, is unlikely under proper fermentation and preservation conditions.

## 4. Effect of Climate Change on Plagues and Diseases

Alterations in climatic conditions can significantly impact the evolution of plagues and diseases, as well as the natural fauna that serve as protective agents in olive groves. This may lead to an escalation in the number of treatments applied to the cultivation, potentially resulting in significant repercussions on the final yields of olive farming systems [128].

The decline in floristic diversity has been shown to lead to a reduction in fauna, disrupt biological control mechanisms, and trigger outbreaks of insect pests [129]. Temperature fluctuations influence the rate of insect multiplication, impacting not only their survival but also the potential for pathogen evolution. For instance, in the summer of 2013, a substantial portion of olive tree cultivation in the Puglia region of Southern Italy was decimated by the infection of *Xylella fastidiosa* [130]. Additionally, there appears to be a growing consensus that the olive fly’s attacks will experience a shift or intensification in “warmer” regions due to the rise in average temperatures during the summer months. This could lead to a delay in the last flight of the olive fly, tempting some olive growers to consider a final treatment close to the fruit harvesting period. As a consequence, recent years have witnessed routine analytical assessments confirming the presence of traces of chemical products in crops [131]. Further research is necessary to ascertain the potential effects of these contaminants on consumer health and their impact on the fermentative process in the case of table olives.

## 5. Economic Impact of Climate Change on the Olive Sector

The magnitude of the economic impact on the table olive sector resulting from climate change is challenging to assess due to the numerous factors. However, according to research conducted by Resco [132], if global temperatures surpass the pre-industrial average by 2 °C before 2050, approximately 80 percent of Andalusia could become inhospitable for some dry-farming olive varieties, including Hojiblanca and Manzanilla. This study estimated that climate change has already reduced Spain’s annual agricultural turnover by 6%, representing a loss of 550 million euros. The annual revenue of the olive sector is approximately 4 billion euros, with 1.5 billion coming from table olives and 2.5 billion from olive oil. The study warns that annual losses are likely to increase according to current climate projections.

Nonetheless, a comprehensive consideration of the economic losses stemming from climate change in the olive sector must account for multiple factors, including market prices. In 2023 alone, forecasts indicate a harvest that is 50 to 60% lower than in 2022 due to droughts [132]. Moreover, economic losses should not be limited to those resulting from olive production in the groves but should also encompass losses due to potential microbiological alterations that may occur in the fermenting process, as well as direct and indirect job losses.

According to the Financial Times in 2023 [133], olive oil prices have been continuously rising, reaching record levels, primarily due to a prolonged period of unusually dry weather in Southern Europe damaging crops. European prices exceeded EUR 4 per kilogram for the first time in September 2022, but they have now surged to over EUR 7 per kilogram due to rising temperatures and a lack of rain in Spain, the world’s largest producer, as well as in Italy and Portugal. In short, the recent trend of increase in consumer’s prices is related to the trend of reduced crop yields and fruit losses as a consequence of climate change.

## 6. Climate Adaptation Measures

### 6.1. Selection of Resilient Varieties

The development of “climate-smart food systems” is required as alerted by Wheeler and Braun (2013) [134]. Olive cultivation is not an exception, and the adoption of varieties better adapted to warmer temperatures and resilient to heat waves, drought, and higher levels of CO_2_ should be taken into consideration.

Selecting olive varieties with higher tolerance to heat, drought, and changing rainfall patterns is crucial in adapting to climate change. One strategy to consider can rely on genetic improvement programs focused on developing olive varieties suited to such future conditions, considering temperature, humidity, and CO_2_ interactions. Another strategy consists of preserving the vast genetic pool of over 2000 olive varieties well-adapted to their regions and well-known for their robustness. Nature-based solutions can be valuable and should be noted for their simplicity and effectiveness. Safeguarding biodiversity is also important from an economic viewpoint. For instance, Cabezas et al. [135] found that adjusting flowering time and using drought-tolerant olive varieties effectively reduce climate change impacts. Zaied and Zouabi (2016) [136] reported decreased olive yields in Tunisia due to climate change, but suggested selecting heat- and drought-resistant varieties to enhance adaptability. So, both strategies can be combined: the selection of autochthonous varieties and their further genetic improvement to enhance adaptation to the upcoming environmental changes towards a more arid climate. Implementing appropriate breeding systems, focusing on high tolerance to water and heat stress, is crucial for this adaptation strategy [137]. Some olive clones/varieties have already shown greater resistance to high temperatures and water stress conditions.

### 6.2. Measures during Fermentation

The uncontrolled growth and metabolism of microbial species during fermentation may be aggravated by global warming. Strategies adapted to each type of table olives will be increasingly needed to limit risks and tackle unpredictable situations, while maintaining their distinctive organoleptic features. The development of tailored starters will allow controlling tailored fermentations, ensuring better efficiency, predictability, consistency, and safety. Starters have already been developed and applied successfully in certain circumstances [55,138,139,140].

Suitable starters should possess adequate technological and safety properties, such as (a) adaptation to the brine’s environment (phenolic content, temperature, and pH); (b) increase in brine acidity through the consumption of sugars and production of organic acids; (c) producing antimicrobial compounds (bacteriocins and killer factors) to inhibit undesirable indigenous microbiota (enterobacteria, clostridia, pseudomonas, and fungi); (d) producing adequate enzymes to contribute to the debittering of olives and improvement of palatable attributes of the fermented final products; (e) binge generally recognized as safe (GRAS); (f) being resistant to phages; and (g) guaranteeing the typicity of the various final products in the various regions, considering the local cultivars [87,141].

Various studies demonstrate the potential of epiphytic LAB strains as natural agents to limit toxigenic fungi growth and metabolism of mycotoxins in table olives [142]. In addition, the adoption of various technological approaches to reduce undesirable microbial growth before storage and packaging will be necessary. This way, table olives’ stability during shelf-life is ensured, in the context of increasing global temperatures, as well as safety.

In this sense, it is mandatory to apply and increase preventive and mitigation measures to reduce the risk of contamination with microbial spoilers and pathogens throughout the supply chain of table olives. Some of these measures are summarized in Table 5.

Regarding table olives, climate change is having an increasing impact on olive phenology and fruit composition, affecting table olive fermentation, final product microbial and chemical quality, organoleptic properties, and shelf-life stability. Some variables’ clear consequences are poorly understood and may depend on the olive variety. The main climate-change-related aspects are advanced harvest times, increased olive sugar levels, and composition of phenolic compounds and flavonoids.

The interactions between intrinsic parameters of olives, epiphytic microbiota, fermentation microorganisms, contamination microbiota, and extrinsic factors are complex and oftentimes unpredictable. If climate change results in alterations of any of these factors, it may have undesired consequences on the hygiene and safety of the table olive supply chain. In addition, microbial species are also adapting, and increased cases of overgrowing, pathogenicity, and mycotoxin production have adverse health effects that are likely to occur in the future. The table olive industry must be vigilant to the need to apply for new hygiene programs. In addition, the reduced availability and quality of the water in the food industry will create new challenges in the management of hygiene. Moreover, water is critical on olive oil processing.

The fermentation of table olives is a crucial process that imparts a distinctive flavor and texture to this product. One of the key factors in controlling this process is pH, as it plays a critical role in inhibiting the growth of pathogenic microorganisms and promoting the activity of beneficial LAB. Thus, maintaining proper pH levels becomes an essential practice to ensure food safety and the quality of the final product. In the case of Spanish-style olives, where pH values can be higher than 10 units, especially at the beginning of fermentation due to the alkali released by the fruits, it is common to use acids at the start of fermentation, primarily hydrochloric acid. However, in other types of preparations, such as natural olives, other acids like acetic, lactic, or citric acid are also used [14,143,144], as well as a mixture of them. In this way, the intention is to adjust the pH to levels below 4.5 to promote the growth of LAB and inhibit pathogenic microorganisms.

In conjunction with pH control, another prevalent industry practice involves introducing glucose into fermentation brines. This practice serves as a preventive measure against the proliferation of spoilage microorganisms, particularly in scenarios where the available fermentable material is inadequate. Glucose serves as an additional source of fermentable sugars for LAB, promoting faster and more controlled fermentation. By providing sufficient fermentable substrates, the growth of beneficial bacteria is encouraged, which in turn inhibits the development of undesirable microorganisms. However, it is important to note that the addition of glucose should be carried out cautiously to avoid imbalances in the microbiota and the flavor profile of the final product. Excessive glucose could promote the growth of undesired microorganisms and alter the organoleptic characteristics of the olives. Moreover, it is common to employ a combination of the aforementioned strategies, such as using glucose and starter cultures of LAB, to either reactivate or guide the fermentation process [145]. Perricone et al. [145] reported an enhancement in the fermentation process by inoculating *L. plantarum* and adding glucose in “Bella di Cerignola” table olives, a traditional variety from the Apulian region in Southern Italy.

On the other hand, the decrease in fruit polyphenol content observed after the high-temperature treatment raises significant implications for the quality and health benefits of the fruits. Polyphenols are bioactive compounds known for their antioxidant properties and potential health benefits. They play a crucial role in protecting the plants from oxidative stress, and contribute to the fruits’ sensory attributes, such as color, flavor, and aroma. Several studies have shown that environmental factors, including temperature, can influence the polyphenol type and content in fruits. For instance, a study by Khayyal et al. [146] on grape berries reported that exposure to high temperatures led to a reduction in anthocyanin and flavonoid levels, which are important subclasses of polyphenols responsible for the red color and health-promoting properties of grapes.

Polyphenols in olives, such as hydroxytyrosol and oleuropein, have been linked to various health benefits, including antioxidant, anti-inflammatory, and cardioprotective effects [147,148]. Therefore, any reduction in polyphenol content due to high-temperature treatment may have implications for the nutritional value and potential health effects of olive products. Moreover, fluctuations in fruit polyphenol content can also impact the organoleptic properties of the fruits, influencing their taste, aroma, and color. Consumers often associate the sensory attributes of fruits with their overall quality, and may prefer products with higher polyphenol content due to their perceived health benefits and better taste. It is important to consider these findings in the context of climate change and the increasing occurrence of extreme temperatures in certain regions. As global temperatures continue to rise, there may be a need to adapt agricultural practices and explore strategies to mitigate the impact of high temperatures on fruit polyphenol content. This could include the selection of olive varieties more resistant to heat stress or implementing agronomic practices that provide shade and cooling for the fruit-bearing trees during periods of extreme heat.

## 7. Conclusions

Climate change will reshape the olive industry, from the cultivation of olive trees to the processing of olive products. To confront the looming challenges, it is imperative to prioritize the selection and preservation of olive varieties that exhibit resilience to the escalating threats of rising temperatures, drought, and altered rainfall patterns. This involves both genetic improvement programs to develop climate-smart varieties and the safeguarding of the existing genetic pool.

As we delve into the intricacies of olive fermentation, we recognize the necessity of tailored approaches to ensure controlled and predictable processes. The development and utilization of starter cultures with specific characteristics has become paramount. These starters should not only adapt to brine environments, but also enhance brine acidity, inhibit undesirable microbiota, and contribute to the debittering and flavor enhancement of olive products.

However, beyond the known challenges lie the uncertainties of the microbial world. Many microorganisms essential to olive fermentation defy cultivation in laboratory settings. This knowledge gap poses significant challenges, particularly in the context of climate change, which may lead to unpredictable shifts in microbial populations and their associated risks. It is crucial to acknowledge that the majority of microorganisms inhabiting the olive ecosystem remain elusive to our understanding. Their roles, interactions, and responses to climate-induced changes remain shrouded in mystery. This knowledge gap highlights the complexity of microbial dynamics in olive processing and calls for more research to bridge this significant lacuna.

As we confront this uncertainty, it is evident that a dual approach of adaptation and mitigation is necessary. Adaptation efforts should focus on breeding for resilience, conserving genetic diversity, and developing climate-smart strategies in olive cultivation and processing. Simultaneously, mitigation measures should be deployed throughout the supply chain to ensure food safety and quality, particularly in the context of evolving climate conditions.

In conclusion, the olive industry stands at a critical juncture, facing unprecedented challenges driven by climate change. The unknown aspects of the microbial world, coupled with the known impacts on olive cultivation and processing, necessitate immediate action. As we move forward, we must prioritize research to unravel the complexities of microbial communities, foster innovation in climate-resilient olive varieties and starter cultures, and implement rigorous adaptation and mitigation strategies. Only through a holistic and proactive approach can we hope to secure the future of this ancient and invaluable industry in a changing world.

## Figures and Tables

**Table 1:** Average temperature (maximum, minimum, and total), rainfall, and final fermentative values of Spanish-style table olives (pH and free acidity) in two different industries, recovered from the Arahal region (Seville, Spain) for 4 years (2019–2022). Values are presented as an average ± standard deviation.

Year	Temperature (°C)			Average Rainfall (L/m^2^/Year)	Fermentation Control Parameters	
	Maximum	Minimum	Average	Rainfall	pH	Free Acidity (%)
2019	25.40 ± 7.14	11.30 ± 5.87	18.35 ± 9.61	0.93 ± 3.99	4.16 ± 0.19	0.90 ± 0.25
2020	25.97 ± 7.76	12.36 ± 5.66	19.16 ± 9.61	1.04 ± 3.52	4.25 ± 0.07	1.01 ± 0.05
2021	26.33 ± 7.86	12.69 ± 5.89	19.51 ± 9.73	1.03 ± 3.64	4.44 ± 0.21	0.85 ± 0.15
2022	27.96 ± 8.01	13.46 ± 5.94	20.71 ± 10.12	0.90 ± 3.61	4.36 ± 0.08	0.76 ± 0.04

**Table 3:** Potential microbial safety hazards during table olive processing as a consequence of climate change.

Microbial Groups of Interest	Occurrence or Survival in Table Olives	Relevant Characteristics That Increase Microbial Risks in the Scenario of Climate Issues
***Bacillus cereus*** (Main source/origin: soil, dust, vegetation)	Survival in Spanish-style Conservolea olives [84].	Heterogeneous species comprising psychrotrophic and mesophilic strains (which grow at 37 °C) that can survive at temperatures below 10 °C. Producer of endospores and biofilms. Spores survive in severe conditions (heating, freezing, drying, and ultraviolet) that generally destroy vegetative cells [85].
***Campylobacter*** Main source/origin: soil, dust, vegetation	Detected in fruit biofilms from commercialized table olive packaging by metataxonomic analysis [86].	Considered thermophilic, *Campylobacter* grows better at high temperatures. It can grow and multiply more rapidly in temperatures between 20 °C and 45 °C. Prolonged storage of unpasteurized foods at inappropriate temperatures, such as within the mentioned range of temperate temperatures, can allow the growth of *Campylobacter* in the food.
***Clostridium* sp. *(C. botulinum*, *C. perfringens)*** Main source/origin: soil, animals, dust, air, water	Part of fruit microbiota at the beginning of fermentation [87]. Associated with table olives and recalls of the suspected products have been reported [88]. Sulphite-reducing *Clostridium* spores were detected in numerous samples of commercialized table olives [88]. *Clostridium* (species not specified) were found in the fermentation of Bella di Cerignola table olives [89].	*C. botulinum*: Physiologically heterogeneous species. Producer of endospores and neurotoxins. Proteolytic strains (PS): Optimum growth temperature 37 °C-42 °C. Minimum growth pH: 4.6 Non-proteolytic strains (NPS): Optimum growth temperature 25 °C. Minimum growth pH: 5 NaCl concentration preventing growth: PS: 10%; NPS 5% PS: Inadequate heat treatment will permit the survival of spores that will germinate/grow and produce neurotoxin during ambient storage. NPS: time and/or temperature abuse of commercial or home-made refrigerated foods [90]. *C. perfringens:* Producer of endospores and endotoxins. Optimal conditions for food poisonings arise when food contaminated with spores is slowly chilled or held in a temperature range of 10–54 °C, allowing germination and rapid growth of *C. perfringens*. Upon ingestion of large numbers of vegetative *C. perfringens* cells, they sporulate in the intestinal lumen and produce endotoxins [91].
**Pathogenic Enterobacteria,** **e.g., *Escherichia coli* O157:O7** Main source/origin: water, animal stools. Fecal matter can contaminate food and water, including irrigation water and recreational water.	Survival in table olives of Halkidiki variety [92]. Survival in table olives of different varieties [93]. Survival in Spanish-style Conservolea olives [60].	*E. coli* grow from 4 °C to 50 °C, with an optimum at 37 °C. The pathogenicity of *E. coli* has been explained by the acquisition of a series of virulence genes [94]. The ability of *E. coli* strains to survive and grow in environments other than the gastrointestinal tract poses a serious public health problem. *E. coli* was isolated from soil, manure, and irrigation water, and demonstrated the ability to colonize the internal compartments of plants [95] and plant roots [96]. Vegetal material can contribute to the dissemination of bacteria in industrial food processing and packaging environments, contributing to their dissemination in the food chain if good manufacturing practices are not respected. *E. coli* O157:H7 has the ability to survive and multiply outside the intestine. In table olives, the elimination of pathogenic *E. coli* is facilitated by a synergy of factors (lactate, phenolics, a_W_, bacteriocins) related to the overgrowth of preselected starter cultures [97,98].
***Listeria monocytogenes*** Main source/origin: soil, animals, insects, compost, decaying vegetation, processing environments. Persist in mammalian and avian feces	Has been found in marketed table olives in Europe, such as Spanish-style process green Nocellara dell’Etna olives [99,100]. Associated with fermented olives with and without starter cultures from different Greek, Italian, and Spanish varieties and fermentation styles [92,99,100]. Survival in table olives of different varieties [94,95,100].	Psychrotrophic growing at temperatures less than 1 °C to 37 °C. Tolerates NaCl concentration up to 10%, pH < 4, as well as phenolic compounds to a certain extent. Producer of biofilms. Data from Caggia (2004) [100] suggest that *L. monocytogenes* grow in fermenting brines and can be more probable when the pH is higher (>4.5) and total phenolic and LAB counts are low. Survival under stressful conditions for long periods (+7 months) was reported by several authors. The ability to survive and grow in multiple habitats is supported by a competent system of adaptation to various stresses [101,102]. The ability to grow at refrigerated temperatures makes this bacteria an actual problem for the food industry and consumers during long storage, even at refrigeration temperatures.
***Salmonella* sp.** Main source/origin: animal stools, human carriers	Survival in black table olive varieties [103]; survival in table olives of different varieties [92,93].	*Salmonella* growth ranges from 5 °C to 47 °C, with an optimum at 37 °C. Numerous studies have demonstrated the survival and growth of *Salmonella* spp. in foods of vegetal origin. *Salmonella* sp. survives well on low a_W_ foods, such as spices and aromatic herbs, which may eventually be used to season table olives. High lipid concentrations seem to have a protective effect on *Salmonella* in low a_W_ conditions [104]. Nascimento et al. [105] warned that high levels of lipids protect *Salmonella* from stomach acidity. Temperature abuses of food products containing *Salmonella* sp. as a result of contamination or cross-contamination may promote its proliferation, especially during storage at inadequate temperatures.
***Enterobacter cloacae***	Isolated from Italian table olives “Bella di Cerignola” [106].	High temperatures, particularly above 30 °C, favored the proliferation of *Enterobacter cloacae*.
***Staphylococcus* sp. and coagulase positive (*S. aureus*)** Main source/origin: human skin, nasal passages, injuries, environment, surfaces	Part of fruit microbiota at the beginning of fermentation. Enumerated in table olive samples from Portuguese open-air markets [107] and Aloreña de Málaga [108]. Detected in the microbial biofilm that covers the fruit, in marketed samples from different varieties and geographical origins of commercialized table olives [86]. Survival in table olives of different varieties [93]. Survival in commercial Aloreña de Málaga table olives [98].	*S. aureus* is one of the most resistant non-spore-forming human pathogens, and can survive for long periods in stressful conditions. Staphylococci are mesophilic. *S. aureus* growth ranges from 7 °C to 47.8 °C, with an optimum at 35 °C. The growth pH range is between 4.5 and 9.3 (optimum 7.0–7.5). Staphylococci are able to grow at low levels of a_W_ (0.83). Strains of *S. aureus* are highly tolerant to salts and sugars. Some strains are resistant to multiple antibiotics and may produce (and attach to) biofilms. Temperature abuses of food products containing *S. aureus* may be responsible for its growth and subsequent production of enterotoxin which can be involved in staphylococcal food poisoning [109].
***Vibrio* sp.** Main source/origin: contaminated waters, salt	Part of microbiota at the beginning of fermentation. Detected in the microbial biofilm that covers the fruit, in marketed samples from different varieties and geographical origins [86].	*Vibrio* growth ranges from 4 °C to 40 °C, with an optimal of 20–30 °C. Growth occurs at NaCl concentrations of 0–10%, with minimum requirements between 1 and 3.5% (halophilic species).
**Bacteriophages** Main source/origin: water, air, vegetables	Viruses that infect strains of *L. plantarum* species from table olive fermentation were isolated from natural green olives [110].	High temperatures and bacteriophages can indirectly select pathogenic bacteria in environmental reservoirs [111].

**Table 4:** Potential microbial spoilage and emerging threats in table olive processing as a consequence of climate change.

Microbial Groups of Interest	Occurrence or Survival in Table Olives	Relevant Characteristics That Increase Microbial Risks in the Scenario of Climate Issues
***Celerinatantimonas* sp.** Main source/origin: salt, salty waters	Associated with the gas pocket formation and quality of Spanish-style green olives [69]. Detected in *Aloreña de Málaga* table olives [97,108].	Growth ranges from 17 to 49 °C, with optimal at 31 °C, and occurs at NaCl concentrations of 2.5–8.0%, with optimal growth at 7.0–7.5% (halophilic species) [69].
***Enterobacteriaceae*** ***E. coli*** (non-pathogenic strains) Main source/origin: water, animal stools	Part of fruit microbiota at the beginning of fermentation. Detected at the beginning of fermentation [71,112]. Coliforms and *E. coli were* enumerated in a few samples of commercialized table olives [107].	Facultative anaerobic group of microorganisms. Enterobacteriaceae are a good indicator of compliance with good hygiene practices. Its presence in fermented/processed olives means that the fermentation occurred inadequately or that contamination occurred after processing. They are also indicators of adequate washing and sanitization in products of vegetable origin and ready-to-eat foods, such as table olives. High numbers of Enterobacteriaceae represent a risk of deterioration due to the production of off-flavors and gas pocket spoilage on the olives’ surface. Some members of this group may contribute to biogenic amine formation [13]. Their survival decreases when starters are used [112]. Some strains may acquire pathogenic genes (pathogenic *E. coli*)
**Fungi (e.g., mycotoxins producers *Aspergillus* sp., *Penicillium* sp., *Alternaria*)** Main source/origin: soil, dust, air, water	Part of fruit microbiota at the beginning of fermentation. Filamentous fungi are found in bulk and packed table olives. They are easily detectable at the surface and/or by the sensorial changes they cause. Its presence can result in product recalls even if the risk ends up being low [113]. *Penicillium* sp. in black table olives [114]. *Alternaria* sp. and *Penicillium* sp. in Greek cultivars [115]. *Penicillium* sp. in packed table olives *Aloreña de Málaga* [97]. *Alternaria* sp. in brined olives from different countries [116]. Mycotoxins: Aflatoxin B1 was reported in black and green olives of Greek origin [117]; Aflatoxin B1 and ochratoxin A were detected in green Italian table olives [118]. Ochratoxin A, citrinin and aflatoxin B1 were detected in natural black table olives of Moroccan origin [119].	Physiologically heterogeneous group globally and ubiquitously distributed, isolated from various habitats. They prefer acidic media (able to grow in pH 2.0) and aerobic conditions, but can also grow in the absence of oxygen. As mesophilic organisms (10–40 °C), however, there are thermotolerant species capable of growing at 50 °C. Climate change is contributing to modifying the geographical distribution of fungi, providing new biotic interactions, food contamination pathways, and production of mycotoxins (neurotoxic and carcinogenic) and infections.
***Pseudomonas* sp.** Main source/origin: ubiquitous, including soil and water.	Detected at different fermentation times in *Aloreña de Málaga* table olives [108,120] and other varieties [86]. Detected in table olive dressing and seasoning material [121]. Detected in commercialized table olive packages [86].	*Pseudomonas*–host interaction could be affected by climate change [122]. Under laboratory conditions, most *Pseudomonas* species grow at an optimal range from 25 to 30 °C. However, some strains can develop in both warm and cold environments, at temperatures between 0 and 45 °C, depending on the species [123].
**Other Emergent risks** Main source/origin: microorganisms that would adapt to climate changes as eukaryotes, prokaryotes, and viruses in biofilms attached to plastic debris/microplastics (MP) impacting ecosystems	[124,125]	Plastic debris is becoming ubiquitous, and certain species are more likely to integrate biofilms in this so-called “plastisphere”. These include eukaryotic (protozoan and helminth) pathogens that may be associated with bacteria, in which there seems to be a tendency for the predominance of human pathogens. The plastisphere can constitute a new niche with a particular microbial ecology that may cause systemic changes [125].

**Table 5:** Preventive/mitigation measures to reduce microbial contamination and growth (pathogens and spoilers) (HACCP—Hazard Analysis and Critical Control Point).

Preventive/Mitigation Measures	Operations/Actions
Raw materials	Hygienic quality of olives and their microenvironment
	Hygienic quality seasonings (herbs and spices, among others)
	Hygienic quality of salt
Use of good quality water	Debittering
	Washing
	Brining
	Preparation of authorized additives and technological auxiliaries
	Disinfection
Fermentation	Select adequate starter mixtures (including LAB and yeasts) to better control fermentation
	Monitor fermentation process: pH, acidity, salt, and microbial hygiene parameters
	Apply corrective measures when needed (brine changes, acidification, salt increase, etc.)
Avoid temperature abuse in the supply chain	Storing
	Transport
	Distribution
	Commercialization
	In restaurants, hotels, and at home
Selection of hygienic-designed equipment and infrastructures	Processing plants with a hygienic architecture,
	Choose hygiene-designed equipment (fermenters, contains, and conveyor belts)
	Fermenters and containers without corners, joints, or right angles
	Filters/incoming air
Selection of cleaning and disinfection plans with the appropriate frequency	Avoid microbial biofilms and limit aerosols development in the industries, equipment, and working surfaces
Adoption of good manufacturing procedures	Prevent contamination and cross-contamination
	Stimulate the adoption of strict personal hygiene
	Provide education for food handlers
	Implement or adapt HACCP principles
Review/adaptation of HACCP principles	Verify critical points
	Review/adapt critical limits
	Check conformity regularly by measuring adequate parameters (pH, microbial, and physicochemical limits)
	Implement corrective measures when a specific critical control point is uncontrolled
	Regular verification
	Maintain updated reports
Fermentative or beneficial microorganisms capable of	Fermentation abilities that produce acids, contribute to the lowering of pH and produce flavor compounds
	Producing antimicrobial compounds (bacteriocins)
	Establishing new biotic interactions
	Internalizing the mesocarp/pulp of the table olives
	Improving the nutritional properties of table olives, notably their vitamin content
	Contributing to the decrease in occurrence of toxins
	Giving origin to probiotic and postbiotic in the final products
